# Liquid Chromatography-Tandem Mass Spectrometry Analysis of Perfluorooctane Sulfonate and Perfluorooctanoic Acid in Fish Fillet Samples

**DOI:** 10.1155/2012/719010

**Published:** 2012-03-18

**Authors:** Viviana Paiano, Elena Fattore, Andrea Carrà, Caterina Generoso, Roberto Fanelli, Renzo Bagnati

**Affiliations:** Department of Environmental Health Sciences, Mario Negri Institute for Pharmacological Research, 20156 Milan, Italy

## Abstract

Perfluorooctane sulfonate (PFOS) and perfluorooctanoic (PFOA) acid are persistent contaminants which can be found in environmental and biological samples. A new and fast analytical method is described here for the analysis of these compounds in the edible part of fish samples. The method uses a simple liquid extraction by sonication, followed by a direct determination using liquid chromatography-tandem mass spectrometry (LC-MS/MS). The linearity of the instrumental response was good, with average regression coefficients of 0.9971 and 0.9979 for PFOS and PFOA, respectively, and the coefficients of variation (CV) of the method ranged from 8% to 20%. Limits of detection (LOD) were 0.04 ng/g for both the analytes and recoveries were 90% for PFOS and 76% for PFOA. The method was applied to samples of homogenized fillets of wild and farmed fish from the Mediterranean Sea. Most of the samples showed little or no contamination by perfluorooctane sulfonate and perfluorooctanoic acid, and the highest concentrations detected among the fish species analyzed were, respectively, 5.96 ng/g and 1.89 ng/g. The developed analytical methodology can be used as a tool to monitor and to assess human exposure to perfluorinated compounds through sea food consumption.

## 1. Introduction

Perfluorinated compounds (PFCs) are a large group of chemicals characterized by a fully fluorinated hydrophobic chain and an hydrophilic head. Such properties, in combination with a high chemical stability, make these compounds unique for their ability to repel both water and oils. Over the last 40 years PFCs have been produced for a large number of applications, such as surface treatments for coatings, clothes, carpets, packaging products, cookware, and food contact papers. Nowadays they are global contaminants which have been detected in environmental and biological samples from different areas worldwide [1–8].

Among these compounds, perfluorooctane sulfonate (PFOS) and perfluorooctanoic acid (PFOA) seem to meet the criteria of persistence, biomagnifications, and long-distance transport to be included in the definition of persistent organic pollutants (POPs), under the Stockholm Convention; moreover they cause particular concern because they have been shown to be carcinogenic in experimental animals [9–11]. Detailed toxicological studies have suggested that peroxisome proliferation, hepatotoxicity, carcinogenicity, immunotoxicity, lipid metabolism, and developmental toxicity may be associated with chemical exposure to PFOS and PFOA [12–15].

Despite the fact that an increasing number of laboratories have developed analytical methods to quantify these emerging pollutants, for several years the quality of the data were a major issue, mainly because of the poor availability of mass-labeled standards, matrix effects and interferences, and blank contamination from labware and instrumentation [16]. As opposed to other POPs, which accumulate in fat-rich tissues, these chemicals are water soluble and tend to bind to serum proteins and to accumulate in liver, kidneys, and bladder of exposed organisms. For this reason most of the work concerning analytical determination of PFOS and PFOA has been done on water and blood samples [17–19] whereas limited information is available regarding the performance of analytical methods to quantify perfluorinated compounds in other environmental samples which can cause human exposure.

Results of intercalibration studies for these compounds showed that the agreement between laboratories was worst for fish tissues, when compared to blood, plasma, liver, and water samples [20] even if significant improvements were recently achieved by using solvent-based calibration curves with mass labeled internal standards [16].

Liquid chromatography—tandem mass spectrometry (LC-MS/MS) is the method of choice for analyzing PFOS and PFOA in environmental and biological matrices. In general, LC-MS/MS is a highly sensitive technique for measuring the concentration of analytes in complex matrices because of a higher signal-to-noise (S/N) ratio, as the result of the fragmentation of specific isolated precursor ions [21].

In this study we present an analytical method based on isotope dilution with ^13^C labeled internal standards and determination by LC-MS/MS. The method quantifies PFOS and PFOA at very low levels in edible part of wild and farmed fish samples, and avoids a solid phase extraction step (SPE), resulting in a lower sample contamination by extraction solvents and in a shortening of the analysis time. The purpose was to provide a sound and reliable methodology to quantify these pollutants in fish, which seem to be one of the main routes of exposure to these pollutants for human population.

## 2. Experimental

### 2.1. Chemicals and Materials

Analytical standards were the following: perfluorooctanoic acid (PFOA), sodium perfluorooctane sulfonate (PFOS), perfluoro-n-[1,2,3,4-^13^C_4_]octanoic acid (^13^C_4_-PFOA), and sodium perfluoro-[1,2,3,4-^13^C_4_]octane sulfonate (^13^C_4_-PFOS). All this chemical were acquired from Wellington Laboratories (Wellington Laboratories Inc., Guelph, Ontario, Canada), in the form of 50 *μ*g/mL methanol solutions, which were stored at −20°C. The solvents and reagents used for sample preparation and chromatographic separation were the following: methanol LC grade, (Carlo Erba, Milan, Italy), ammonium acetate, LC-MS grade, (Fluka, Buchs, Switzerland), acetonitrile, LC grade (Carlo Erba), and ultrapure Milli-Q water, obtained with a Milli-Q RO Plus 90 apparatus (Millipore, Molsheim, France).

### 2.2. Sample Preparation

Aliquots of homogenated fish muscles (1 g) were weighed in glass tubes (10 mL) and then mixed with 2 mL of methanol, containing 1 ng of each IS (^13^C_4_-PFOA and ^13^C_4_-PFOS). The tubes were vortexed for 1 min and ultrasonicated for 40 minutes to improve the diffusion of standards and analytes. The samples were then centrifuged for 10 minutes at 2800 rpm, and 0.5 mL of the supernatants were transferred into 1.5 mL glass vials for instrumental analysis. Before the analytical run, 0.5 mL of Milli-Q water were added to the vials to obtain a 1 : 1 methanol : water solution suitable for LC injection.

### 2.3. Liquid Chromatography—Tandem Mass Spectrometry

Samples were analyzed with an LC system coupled to a triple quadrupole mass spectrometer. The methanol/water extracts (40 *μ*L injection volume) were chromatographed on a C18 XTerra MS column (2.1 × 100 mm, 3.5 *μ*m), using an LC system which consisted of two 1200 Series pumps and autosampler (Agilent, Foster City, CA). The mass spectrometer system was a 6410 triple quadrupole equipped with an ESI source, operated in negative ion mode (Agilent, Foster City, CA). The ESI nebulizer gas temperature was fixed at 300°C, at a flow rate of 8 mL/min and a pressure of 40 psi; the ESI capillary voltage was 2800 V. The detailed instrumental conditions for every analyte are reported in Table 1.

The mobile phase consisted of 5 mM ammonium acetate in water (solvent A) and acetonitrile (solvent B) at a flow rate of 0.2 mL/min. The elution gradient started from 36% to 56% of solvent B in 12 min and was increased to 99% of B in 1 min. The total run time was 13 min, followed by an equilibration time of 7 min between every injection.

PFOS and PFOA were quantified by multiple reaction monitoring (MRM) using the most abundant precursor/product ion transitions (499 → 80 for PFOS and 413 → 369 for PFOA) whereas the second MRM transitions (499 → 99 for PFOS and 413 → 169 for PFOA) were used as qualifiers. Retention times were compared with reference standards to identify each compound. Eight standard solutions containing different amounts of analytes (0, 0.04, 0.1, 0.2, 0.4, 1, 2, and 4 ng) and a fixed amount of each IS (0.5 ng), were injected to obtain calibration curves for each substance. Blank samples containing only extraction solvents and IS were included in all analysis batches.

Instrumental detection limits (IDL) and instrumental quantification limits (IQL) were determined by direct injection of decreasing amounts of each substance. Detection limits (LOD) quantification limits (LOQ) for the whole method were calculated by analyzing in triplicate different samples of fish homogenates. The LOD and IDL were calculated as the concentrations giving peaks with a signal-to-noise ratio equal to 3 while the IQL and the LOQ were calculated as the concentrations giving signal-to-noise ratios equal to 10.

The recoveries, expressed in percentage, were estimated by analyzing in triplicate blank fish samples spiked with different amounts of analytes (0.2, 1, 4 ng/g). Fixed amounts of internal standards (0.5 ng/g) were added after the extraction procedure and before transferring the methanol aliquots to the autosampler vials. The repeatability of the method was determined in the same and in three separate days, by analyzing in triplicate three real samples in which we found different amounts of PFOA (0.12, 0.15, and 0.20 ng/g) and PFOS (0.80, 0.41, and 0.67 ng/g).

## 3. Results and Discussion

### 3.1. Liquid-Phase Extraction

At the beginning of the method development, SPE cartridges (Evolute ABN) were used for the extraction of PFOS and PFOA from fish samples, but the results were unsatisfactory. Recoveries were low and frequent contaminations of blank samples were observed (especially for PFOA), probably because of contaminated solvents, or PTFE tubings or caps. For these reasons a new sample preparation procedure, consisting of a liquid-phase extraction only, followed by a direct instrumental analysis, was developed.

### 3.2. Liquid Chromatography-Tandem Mass Spectrometry

**Figure 1:**
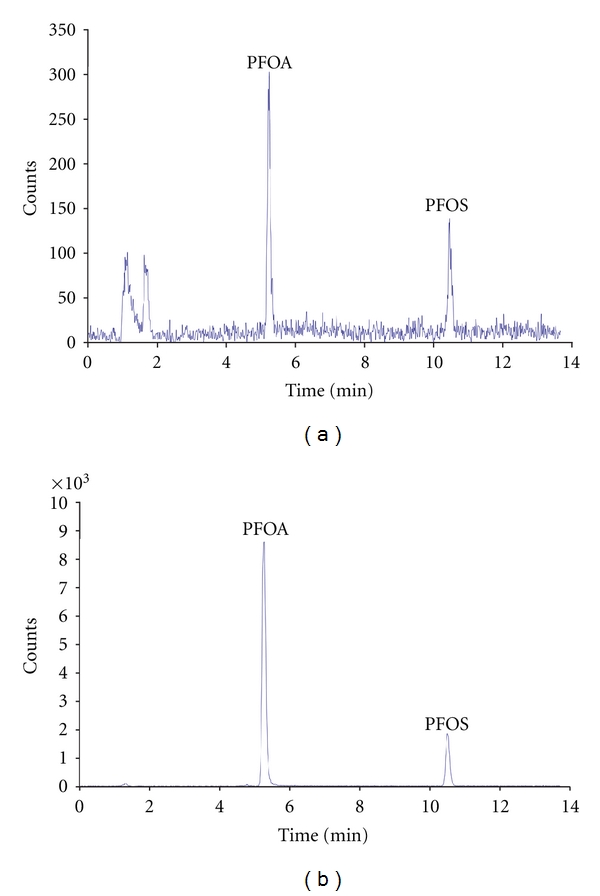
MRM total ion chromatogram for (a) extracted fish sample and (b) standard solution.

As described above, the compounds were analyzed by LC-MS in MRM mode and quantified with an isotope dilution method. Matrix effects were minimized using this approach, because ion suppressions or enhancements may be considered equivalent for analytes and their ^13^C labeled analogues. Examples of MRM chromatograms are reported in Figure 1 for an extracted fish sample and a standard solution.

Results for linearity, repeatability, recoveries, and sensitivities of the method are reported in Table 2.

### 3.3. Validation Data

The range of linearity of the instrumental response was assumed to be the same of the calibration curves (0–4 ng). Intraday and interday repeatability of the method for both analytes was evaluated by analyzing three different samples in triplicate in the same day and in three different days. In Table 2 the repeatability is expressed as average coefficient of variation.

Instrumental detection limits (low pg range) and limits of detection of the whole method (sub-ng/g range) were similar for both the analytes. Berger and Haukås [22] showed in their work lower IDL values (IDL_PFOS_ =  0.3 and IDL_PFOA_ =  1 pg injected). This is probably due to a different instrumental technique based on LC coupled to a time-of-flight (TOF) mass spectrometer. They quantified perfluorinated compounds using the precursor ion of each substance whereas, as described above, we quantified the compounds by MRM using the two most abundant precursor and product ion transition; this led us to develop a methodology with a higher specificity. Furthermore, the comparison between our data and those reported in another investigation carried out by Nania et al. [23], where the contamination levels of PFOS and PFOA were evaluated in the edible fish of the Mediterranean Sea, shows that LODs given in Table 2 are lower, even though these values were derived differently in the two works. Finally, the LOD values of this method are similar to those calculated by Ericson et al. [24] in a study concerning human exposure to perfluorinated chemicals through seafood consumption, for the determination of the dietary intake of these compounds by the population of Tarragona County (Catalunia, Spain).

### 3.4. Application to Real Samples

The analytical methodology developed in this study was applied to quantify PFOS and PFOA in 65 real samples, in particular in homogenized fillets of wild and farmed fishes from the Mediterranean Sea.  Table 3 shows the concentration levels (mean ± standard deviation) of PFOS and PFOA that we found in wild and farmed fishes.

Generally very low concentrations of these pollutants were found in farmed fishes whereas the highest concentrations of PFOS and PFOA detected among the wild species analyzed were, respectively, 5.96 ng/g in an anchovy (*Engraulis encrasicolus*) and 1.89 ng/g in a Norway lobster (*Nephrops norvegicus*). As written above, the LODs of the method here described are among the lowest reported in the literature. This is a requirement for the quantitation of PFOS and PFOA in the low ng/g range, as in the samples of Mediterranean fishes we have analyzed. In other studies, several fish species from different geographical areas were analyzed and the levels of these contaminants were significantly higher [25–28]. In a recent paper, Delinsky et al. [29] quantified perfluorinated compounds in bluegill sunfish (*Lepomis macrochirus*) fillet samples, collected from selected areas of Minnesota and North Carolina, reporting PFOS concentrations in the range of 2.08–275 ng/g.

## 4. Conclusions

A new methodological approach was developed to quantify PFOS and PFOA in the edible part of wild and farmed fishes from the Mediterranean Sea. This method allowed measurements in the low ng/g range, with high recoveries and a good intraday and interday repeatability.

These two contaminants were found in our samples in lower concentrations than those reported in other studies, in which biological and environmental samples from different geographical areas were analyzed.

PFOS and PFOA are ubiquitous persistent organic pollutants with possible environmental and human health risks. The analytical method here developed can be used for the determination of these substances in a large number of fish samples in a short time. The data collected in this way could be useful to provide a basis for a more complete risk assessment of human exposure to perfluorinated compounds through sea food consumption.

## Figures and Tables

**Table 1:** Ionization and fragmentation conditions for PFOS and PFOA, using an ESI source in negative ion mode.

Compound name	Precursor ion (Da)	Product ion (Da)	Dwell time (ms)	Fragmentor energy (V)	Collision energy (eV)
^13^C_4_-PFOS	503	99	100	200	48
^13^C_4_-PFOS	503	80	100	200	56
PFOS	499	99	100	200	48
PFOS	499	80	**100**	200	56
^13^C_4_-PFOA	417	372	100	80	4
^13^C_4_-PFOA	417	172	100	80	16
PFOA	413	369	100	80	4
PFOA	413	169	100	80	16

**Table 2:** Method linearity as average regression coefficient (ARC), instrumental detection limit (IDL), instrumental quantification limit (IQL), limit of detection (LOD) and limit of quantification (LOQ), recoveries (mean ± standard deviation, *n* = 3) and Interday and Intraday repeatability as average coefficient of variation (CV%), for PFOS and PFOA.

	PFOS	PFOA
ARC	0.9971	0.9971
IDL (pg injected)	4.40	2.41
IQL (pg injected)	14.70	8.03
LOD (ng/g)	0.04	0.04
Recovery (%)	90 ± 9.6	76 ± 5.5
Interday CV% (mean)	14	20
Intraday CV% (mean)	8	19

**Table 3:** Concentration levels (mean ± standard deviation) of PFOS and PFOA in wild and farmed fish samples.

Samples	*n*	Concentration (ng/g)	
		PFOS	PFOA
Wild fish	52	1.24 ± 1.10	0.19 ± 0.35
Farmed fish	13	0.05 ± 0.01	<0.05
